# Editorial: Integrative approaches to fibromyalgia: advancing multidisciplinary management strategies

**DOI:** 10.3389/fmed.2026.1863815

**Published:** 2026-05-15

**Authors:** Angelo Alito, Jacopo Maria Fontana

**Affiliations:** 1 Department of Biomedical, Dental Sciences and Morphological and Functional Imaging, University of Messina, Messina, Italy; 2 Brain Mapping Lab, Department of Biomedical, Dental Sciences and Morphological and Functional Imaging, University of Messina, Messina, Italy; 3 IRCCS, Istituto Auxologico Italiano, Verbania, Italy

**Keywords:** central sensitization, fibromyalgia, integrative medicine, metabolic profiling, multidisciplinary management, patient adherence, rehabilitation technology

Fibromyalgia (FM) is one of the most challenging conditions in modern medicine. Defined by widespread pain, cognitive “fog,” and persistent fatigue, it does not respond well to traditional “one-size-fits-all” approaches (1, 2). However, over the last decade, our understanding has shifted, and we now view FM as a systemic phenomenon of central sensitization, requiring a deeply human, multidisciplinary response rather than merely suppressing the symptoms (3, 4). This acknowledges that FM is not a static disorder, but rather a dynamic state in which the nervous, endocrine, and immune systems interact in a complex feedback loop (5, 6). This Research Topic, “*Integrative approaches to fibromyalgia: advancing multidisciplinary management strategies*,” brings together contributions that are shifting the field from reactive management toward proactive, precision-based, integrative care. By integrating different therapeutic perspectives, we aim to address the multifaceted nature of the syndrome and move beyond the limitations of monotherapy to a more comprehensive, patient-centered paradigm that acknowledges the biological complexity of chronic pain.

## Human data: translating silence into evidence

A significant barrier to effective FM care has always been the “invisibility” of patients' suffering, which often leads to diagnostic delays and clinical uncertainty. The study of Úbeda-D'Ocasar et al. addresses this issue by examining the complex relationship between pain, physical functionality, and body composition. Their findings remind us that functional disability is not merely a consequence of pain, but rather a consistent issue that necessitates early, comprehensive evaluation to restore patients' dignity and quality of life. This perspective is essential for clinicians to recognize that physical limitations often precede or exacerbate psychological distress, creating a debilitating cycle that requires immediate, empathetic intervention.

Furthering this quest for objectivity, Kandakurti et al. use digital inclinometry and posturography to map proprioceptive deficits at the joint level. By converting subjective reports of instability into quantifiable data, the study enables clinicians to develop rehabilitation programs that specifically target balance, shifting the focus from general recommendations to precise physical therapy. This technological integration is crucial; it provides a “visible” map of a “hidden” deficit, allowing for a tailored approach that reduces the risk of falls and improves daily motor confidence. By grounding therapy in measurable biomechanical markers, we can offer patients not just treatment, but a clear, evidence-based path toward reclaiming their physical autonomy.

## The hurdle of adherence: bridging the gap

One of the most concerning aspects of FM management is the low rate of treatment adherence. Patients often find themselves trapped in a cycle of “trial and error,” where side effects or the slow onset of benefits lead to frustration and abandonment of care. The systematic review by Wang et al. confirms that aerobic exercise is a powerful biological modifier, yet we must ask: how can a patient in constant pain maintain such a demanding regimen? The difficulty in adhering to active physical protocols is a clinical reality that we cannot ignore, as the very effort required to heal often exacerbates the patient's baseline exhaustion.

This is where innovation steps in to provide a necessary bridge. Lara-Reyes et al. demonstrate that combining technologies such as pulsed electromagnetic fields (PEMFs) and ozone therapy can offer a “head start,” reducing pain levels sufficiently to allow even the most refractory patients to eventually engage in more active therapies. By acknowledging the patient's immediate need for relief through these advanced modalities, we can foster a stronger therapeutic alliance and improve long-term adherence to the more demanding pillars of multidisciplinary care.

## The metabolic frontier: a personal path

The future of FM care lies in exploring the gut-brain axis and metabolic profiling. Castro Corredor et al. leverage machine learning to demonstrate that a patient's metabolic profile, including conditions such as dyslipidemia, can predict their response to para probiotic supplementation. This emphasizes a key point: The “FM patient” is not a homogeneous entity. Instead, we are dealing with a spectrum of metabolic subtypes that respond differently to identical stimuli.

By identifying baseline factors through advanced algorithms, we can finally move away from the “trial and error” approach. Understanding that a patient's lipid profile or microbiome state can dictate the success of a nutritional intervention allows for a more “surgical” precision in non-pharmacological prescriptions. This data-driven stratification is essential to transform FM management from a generalized protocol into a truly personalized path, where interventions are as unique as each patient's biological and metabolic signature.

## A call for radical collaboration

As Topic Editors, we are encouraged by the shift toward a more humanized science—one that uses high-tech tools to help us understand patients' unique struggles more deeply, rather than distancing us from them. The integration of bioengineering for biomechanical assessment, digital tools for monitoring, and AI for metabolic prediction must serve a single goal: restoring the patient's agency over their own health.

The synergy between bioengineering, nutrition, and physical medicine is not just an academic ideal; it is a clinical necessity and an ethical imperative. For too long, the lack of objective markers has led to the stigmatization of FM patients, often leaving them feeling invisible within the healthcare system. The multidisciplinary strategies presented in this Research Topic provide the framework for a “radical collaboration” where technology and empathy coexist. By bridging the gap between innovative research and clinical reality, we can ensure that patients will no longer feel abandoned by a system that has struggled to see the complexity of their pain for too long. This integrative path is the only way to build a future where every FM patient receives a treatment plan that is not only evidence-based but also deeply respectful of their individual experience.
